# Non alcoholic fatty liver disease in a Nigerian population with type II diabetes mellitus

**DOI:** 10.11604/pamj.2016.24.20.8181

**Published:** 2016-05-06

**Authors:** Titilola Osawaye Olusanya, Olufunmilayo Adenike Lesi, Adekunle Ayokunle Adeyomoye, Olufemi Adetola Fasanmade

**Affiliations:** 1 Gastroenterology and Hepatology Unit, Lagos Nigeria; 2 Endocrine and Metabolism Unit, Lagos Nigeria, Department of Medicine, Lagos Nigeria; 3 Department of Radiology, Lagos, Nigeria, Faculty of Clinical Sciences, College of Medicine, University of Lagos Teaching Hospital, PMB 12003, Idi-Araba, Lagos Nigeria

**Keywords:** Fatty liver disease, ultrasound scan, dyslipidaemia, Africa

## Abstract

**Introduction:**

Worldwide, Non-alcoholic fatty liver disease (NAFLD) has become an important cause of chronic liver disease and cardiovascular morbidity, even more so in subjects with Type II Diabetes Mellitus (T2DM). The aim of this study was to determine the prevalence and risk factors of NAFLD in an African population with Type II Diabetes Mellitus.

**Methods:**

We performed a case control study and evaluated anthropometric and biochemical risk factors for NAFLD in 336 subjects (T2DM and non-diabetic controls). Parameters assessed included estimation of BMI (Body Mass Index), measurement of waist circumference (WC), serum cholesterol including HDL-C, LDL-C and triglyceride and serum transaminases (ALT and AST). Hepatitis B and C viral antibody screening was also performed. The diagnosis of NAFLD was confirmed by identification of hepatic steatosis on abdominal ultrasound scan evaluation and exclusion of significant alcohol consumption.

**Results:**

NAFLD was identified in 16.7% (28 of 168) patients with T2DM compared with 1.2% (2 of 168) non-diabetic controls (Odds Ratio 16.6; p < 0.001). Central obesity (WC > 102cm) and dyslipidaemia (HDL-c < 40mg/dl) were independently associated with NAFLD in male subjects with T2DM (p = 0.03 and p = 0.04 respectively).

**Conclusion:**

NAFLD occurred more frequently in patients with T2DM than controls and was associated with central obesity and dyslipidaemia. The diabetic subjects with NAFLD will require more intensive therapy to decrease the risk of hepatic, cardiovascular and other adverse events.

## Introduction

Non-alcoholic fatty liver disease (NAFLD) is a spectrum of clinical and pathological conditions characterized by excessive lipid deposition in the liver parenchyma in the absence of significant alcohol consumption [1]. In the past decade, NAFLD has gained worldwide prominence as an important public health condition because of the enhanced risk of progressive end-stage liver disease, liver failure and hepatocellular carcinoma [2, 3]. Current evidence suggests that NAFLD is the hepatic component of the metabolic syndrome, a syndrome that also includes central obesity, dyslipidemia, hypertension and insulin resistance/impaired glucose tolerance [3–5]. Recently published cross-sectional and prospective cohort studies suggest that 19% to 30% of asymptomatic adults in the USA and Europe have NAFLD [6, 7]. The prevalence of NAFLD among people with Type II Diabetes Mellitus (T2DM) is significantly higher than reported in the general population and ranges from 21%-78% [4, 8–10]. In T2DM, NAFLD pursues a more aggressive clinical course with necro-inflammation and fibrosis and progression to end-stage chronic liver disease. Beyond these hepatic consequences, NAFLD is being increasingly associated with an excess risk of cardiovascular disease, coronary artery disease, as well as peripheral vascular disease [11–13]. In African populations, there is a dearth of evidence on the prevalence and characteristics of NAFLD. Kruger et al [14], histologically confirmed NAFLD in 45% of an obese population in Western Cape, South Africa. They convincingly demonstrated a strong association between obesity, NAFLD and insulin resistance. This study was the first to describe the clinical characteristics of NAFLD in South Africa, using a study population comprised mostly of Caucasian subjects with only a minority of Africans. There however remains a dearth of epidemiological and clinical evidence on NAFLD in black Africans. The aim of this study was to determine the prevalence and risk factors for NAFLD in black Africans with T2DM. The findings from this study will provide an opportunity to enhance awareness of this condition and contribute to the evaluation and clinical management of the estimated 5 million people with T2DM in Nigeria [15].

## Methods

The study was conducted at a University Teaching hospital in Lagos, South-West, Nigeria. The hospital is a 760-bed federal tertiary referral centre providing both medical and surgical care to the general population of Lagos. Consecutively presenting adults >18 years old with diabetes were evaluated at the endocrine clinic between November 2011 and January 2012. Included were patients with established T2DM diagnosed according to the American Diabetes Association (ADA) diagnostic criteria [16] (ie symptoms of diabetes plus random plasma glucose concentration ≥ 200 mg/dl or fasting Plasma Glucose (FPG) ≥ 126 mg/dl). Controls were age and sex matched, non- diabetic patients with no history suggestive of diabetes eg polyuria, polydipsia, unintentional weight loss and fasting blood sugar level less than 126 mg/dl. Exclusion criteria included alcohol intake in excess of 20 grams per day, history of jejunoileal bypass surgery or extensive small bowel resection, usage of drugs known to cause secondary steatosis like corticosteroids, methotrexate, and amiodarone. Pregnant women and subjects with severe co- morbidities such as malignancies, congestive heart failure and chronic kidney disease were excluded. A structured questionnaire was administered to each subject to obtain information such as bio-data, alcohol intake, current medications as well as family history of diabetes and liver disease. All patients were clinically evaluated and weight (kg) and height (m) were measured. The Body Mass Index (BMI = weight (kg) /height (m2) was calculated and classified based on World Health Organisation (WHO) criteria [17]. Waist circumference was measured from the right side in the mid-axillary line, midway between the lower margin of the least palpable rib and the top of the iliac crest (highest point of the hip bone on the right side) as the point of reference. Hip circumference measurement (HC) in centimetres was done at the point of widest circumference of the buttocks with the tape parallel to the floor. Both waist and hip circumference was taken twice and the average was calculated [18]. Central obesity was defined as waist circumference (WC) more than 88 cm in females and more than 102 cm in males. The average of three BP readings was calculated. Hypertension was considered at a blood pressure reading of more than or equal to 140/90 mmHg or current intake of anti hypertensive or both [19].

Blood samples were taken for evaluation of serum alanine transaminase (ALT), aspartate transaminase (AST), fasting lipid profile including total cholesterol (TCHOL), high-density lipoprotein cholesterol (HDL-C), triglyceride (TG) (Randox Laboratory Limited, UK). Low-density lipoprotein cholesterol (LDL-C) was calculated using Friedwald formulae LDL= (TCHOL-HDL-C) -TG/5) [20]. Dyslipidemia was defined as having one or more of the criteria: LDL-C ≥100 mg/dL, total cholesterol ≥200 mg/dL, triglycerides ≥150 mg/dL, or HDL-C < 40 mg/dL in males and <50 mg/dL in females [19]. Samples were also analysed for detection of anti-hepatitis C virus and hepatitis B surface antigen (Diaspot, USA). Abdominal Ultrasound scan (USS) evaluation was performed (Aloka Pro-sound 3500, Japan) using a 3.75 MHz probe. Findings suggestive of hepatic steatosis included “bright liver” with increased echogenicity, hepato-renal contrast and attenuation of the diaphragm [20, 21]. The diagnosis of NAFLD was based on the absence of alcohol consumption (or alcohol intake less than 20 grams per day) and the presence of hepatic steatosis on USS evaluation. Ethical approval was obtained from the Ethics and Research committee of Lagos University Teaching Hospital Idi- Araba, Lagos, Nigeria prior to commencement of the study. Pre study counselling was done and written informed consent was obtained from all patients prior to enrolment. Minimal sample size was determined using the Cochrane statistical formula [22]. Total study population was estimated to be 320 subjects. Data analysis was performed using SPSS Version 20 (SPSS Inc, Chicago, IL USA). Cases and controls were compared using Chi-square or Fisher's exact test to assess significance between proportions and Students t-test to assess significance with quantitative data. Variables associated with NAFLD, with a significant P-value < 0.05 in univariate analysis, were included in a multivariable logistic regression. Relationships were assesses by Odds ratio (OR) and 95% confidence interval (CI).

## Results

### Characteristics of study subjects

A total of 340 subjects were recruited of which 336 subjects (168 subjects with T2DM and 168 control subjects without T2DM) were included in data analysis. 4 subjects were excluded due to incomplete laboratory evaluation. The mean age (standard deviation) of the patients with T2DM was 53.2 (8.6) years. There were 109 (64.9%) female and 59 (35.1%) males, with a Male to Female ratio of 1: 1.8. Most of the T2DM subjects (60%, n = 101) had only basic education and 88% (n = 148) earned an income of less than three hundred US dollars ($300)/month). There was no significant difference between the cases and control subjects in terms of age, gender, educational background and monthly income. The T2DM subjects were however significantly more likely to have concurrent hypertension (n = 80, 47.6%) and a family history of diabetes (n = 74, 44%) compared to the non-diabetic controls (Odds Ratio 3.2, p < 0.001 and Odds ratio 6.5, p < 0.001 respectively). The socio demographic variables of the study population are shown in Table 1.

**Table 1:** Socio demographic variables of T2DM subjects and controls

VARIABLES	T2DM Subjects n = 168 (%)	Controls n = 168(%)	P-Value
**AGE mean, years (SD)**	53.17±8.6	52.01±8.9	0.1
**Gender**			0.11
Females	109 (64.9)	97 (57.7)	
Males	59 (35.1)	71 (42.3)	
**Level of Education**			0.08
None	4 (2.4)	3 (1.8)	
Primary	43 (25.6)	28 (16.7)	
Secondary	58 (34.5)	73 (43.5)	
Tertiary*	63 (37.5)	64 (38.1)	
**Monthly income(US $)**			0.10
<300	148(88.1)	149 (88.7)	
>300	20 (11.9)	19 (11.3)	
**Concurrent**			<0.001^a^
Hypertension	80 (47.6)	37(22.0)	
Family history of DM	74(44.0)	18(10.7)	<0.001^b^

* Inclusive of University, Polytechnic, Teacher training college

a Odds Ratio 3.2, 95% Confidence Interval 2.00-5.17

b Odds Ratio 6.5, 95% Confidence 1nterval 3.6-11.7

### Anthropometric, biochemical and ultrasound characteristics of study participants

As shown in Table 2, subjects with T2DM were significantly more likely to be overweight and have higher waist/hip ratio than the controls (p = 0.02 and p < 0.001 respectively). The mean systolic blood pressure was significantly higher among the cases compared with the controls (p < 0.001). T2DM subjects had a higher prevalence of dyslipidaeamia with mean total serum cholesterol and LDL-cholesterol significantly higher compared to the control subjects (p < 0.001 and p < 0.001 respectively). Although the mean AST and ALT values appeared higher in diabetics than controls, this association failed to reach statistical significance (p = 0.05 and p = 0.17 respectively). The distribution of clinical and laboratory characteristics of the study participants is shown in Table 2. Antibodies to Hepatitis C virus (anti HCV) were negative in all the study participants. Seven (7) subjects however were positive for Hepatitis B virus. Hepatic steatosis was detected on ultrasound scan evaluation in 28 of 168 (16.7%) T2DM subjects compared to 2 of 168 (1.2%) control subjects. (Odds Ratio 16.6, p < 0.001). These subjects did not have a history of alcohol use (or consumed less than 20 grams of alcohol per day) and thus fulfilled the criteria for NAFLD.

**Table 2:** Comparison of anthropometric, laboratory and ultrasound scan parameters between T2DM patients and control subjects

VARIABLE	T2DM subjects Mean±SD, (n = 168)	Control subjects Mean±SD, (n = 168)	p-value
**BMI (kg/ m^2^)**	28.47±4.39	27.42±5.09	0.02
**Waist Hip Ratio;**			
**Females**	0.93±0.07	0.89±0.07	0.00
**Males**	0.94±0.06	0.89±0.07	0.00
**Systolic BP (mmHg)**	130.41±18.99	123.05±17.74	0.00
**Diastolic BP (mmHg)**	80.59±12.87	79.92±11.54	0.30
**AST (IU/L)**	6.49±3.41	5.86± 3.70	0.05
**ALT (IU/L)**	9.07± 4.34	8.43±7.59	0.17
**TCHOL (mg/dl)**	179.49±40.89	170 ±31.69.	0.00
**LDL-C (mg/dl)**	94.46±40.57	81.26±32.94	0.00
**HDL-C (mg/dl)**	71.07 ±25.25	73.89±23.32	0.14
**Triglyceride (mg/dl)**	75.22±37.96	80.07±31.47	0.10
**Hepatic steatosis Identified on USS (n,%)**	28 (16.7%)	2 (1.2%)	0.00*

SD standard deviation, AST, aspartate aminotransferase, ALT alanine aminotransferase, TCHOL total cholesterol, LDL-C Low density lipoprotein, HDL-C high density cholesterol, USS ultrasound scan.

* Odds Ratio, 16.6; 95% CI (3.9-70.9)

### Characteristics of NAFLD among subjects with T2DM

The characteristics of NAFLD in the diabetic subjects are shown in Table 3. NAFLD occurred more frequently in men (n = 12/59, 20.3%) than in women (n = 16/109, 14.7%). Subjects with NAFLD also appeared to have higher levels of ALT, AST and fasting serum triglyceride than subjects without NAFLD. These differences however did not attain statistical significance (p = 0.53, p = 0.4 and p = 0.06 respectively). A high frequency of obesity (BMI ≥ 30 kg/m^2^) and central obesity (WC >88 cm) was noted in women with T2DM regardless of NAFLD status (p = 0.19 and p = 0.35 respectively). In contrast, diabetic men with NAFLD had a significantly higher prevalence of generalized obesity (BMI ≥ 30 kg/m^2^) than those without NAFLD (58.3% vs. 25.5%; Odds Ratio 4.0; 95% CI; 1.1-15.3). These men also had an 8-fold risk of central obesity with WC >102 cm (9 of 12 subjects, 75%) and a higher prevalence of dyslipidaemia (HDL-c <40 mg/dl in 4 of 12 subjects, 33.3%) than T2DM men without NAFLD (p = 0.002 and p = 0.013 respectively) as shown in Table 3.

**Table 3:** Univariate analysis of the clinical and biochemical factors associated with NAFLD among subjects with T2DM

Variables	ALL N = 168	NAFLD Positive N = 28 (%)	NAFLD Negative N = 140 (%)	P VALUE
**Mean Age, yrs ±SD**	53.17 ± 8.6	53.1±8.3	53.2±8.7	0.98
**Gender**				0.4
**Male:**	59 (35.1)	12 (20.3)	47 (79.6)	
**Female**	109 (64.9)	16 (14.7)	93 (85)	
**BMI female (**≥**30 kg/m^2^)**	42 (38.5)	9 (56.3)	33 (35.5)	0.19
**BMI Male (**≥**30 kg/m** ^**2**^ **)**	19 (32.2)	7 (58.3)	12 (25.5)	0.04*
**WC Males > 102cm**	21 (35.6)	9(75)	12 (25.5)	0.002**
**WC Females >88cm**	84 (77.1)	14 (87.5)	70 (75.3)	0.35
**AST >12 IU/L**	8 (4.8)	2(7.1)	6(4.3)	0.40
**ALT > 12 IU/L**	16 (9.5)	3(10.7)	13(9.3)	0.53
**T-CHOL** ≥ **200 mg/dl**	46 (27.4)	5(17.9)	41(29.3)	0.16
**HDL-c**				
**Female <50 mg/dl**	30 (28.3)	5 (31.3)	25 (26.9)	0.76
**Male <40 mg/dl**	6 (10.2)	4 (33.3)	2 (4.3)	0.013
**LDL-c** ≥ **100 mg/dl**	76 (45.2)	15(53.6)	61(43.6)	0.22
**Triglyceride >150mg/dl**	10 (5.9)	4 (14.3)	6 (4.3)	0.06

WC waist circumference, BMI Body mass index, AST aspartate aminotransferase, ALT alanine aminotransferase, T-CHOL total cholesterol, LDL-c Low density lipoprotein, HDL-c high density lipoprotein-cholesterol. NAFLD non-alcoholic fatty liver disease.

* Odds ratio 4; 95% CI; 1.1-15.3

** Odds ratio 8.7; 95% CI:2.1-37

## Discussion

In this study, we have shown that up to 16.7% (one in six) of the subjects with T2DM had NAFLD, a 16-fold risk of NAFLD compared to non-diabetic controls. Our finding of NAFLD in both the subjects with T2DM and non-diabetic controls corroborates a prior survey from West Africa that reports a prevalence of NAFLD in 9.5% of diabetics and 4.5% of non-diabetic controls [23]. There is however a paucity of evidence in this region. A significantly higher prevalence of NAFLD ranging from 40% to 60% of subjects with T2DM has been reported in Caucasian [11], Indian [24, 25] and Asian populations [26]. In the USA, the prevalence of NAFLD in the general population varies from 15% in Caucasians to 28% in Hispanics [6]. The comparatively lower prevalence of NAFLD in our study may be a reflection of the different socio-demographic and economic characteristic of this African population. The association of obesity and increasing BMI is an established risk factor for NAFLD worldwide. In this current study, the mean body mass index (BMI) and income characteristics suggest that this population is not significantly obese and appear less affluent than western populations. This is not surprising as subjects were recruited from a large urban public hospital in a resource-limited setting. In addition, diagnostic tools used for evaluation of hepatic steatosis have differing accuracy in estimating hepatic fat. The computerized tomography (CT) scan and magnetic resonance imaging (MRI), although more sensitive in detecting minimal levels of hepatic steatosis, are expensive and impractical as screening tools [20, 21]. The ultrasound scan (USS) has greater than 90% sensitivity and specificity in diagnosing hepatic steatosis and was used in this study. It remains the screening modality of choice because of low cost, safety, and accessibility [21]. Another important reason for the differing distribution of NAFLD appears to be related to the racial and ethnic characteristics of the population. In the US Multi-ethnic Study of Atherosclerosis (MESA), the prevalence of NAFLD in African Americans was found to be significantly lower than in Caucasian and Asian populations [6]. Various other studies corroborate this ethnic disparity [27–29]. Recent evidence from prospective multi-ethnic cohort studies have identified a strong association between NAFLD and genetic mutation in several genes including lipolytic enzymes, patatin like phospholipase (PNPLA-3) domain, neurocan gene NCAN and glucokinase regulatory protein gene GCKR in non-Hispanic whites and subjects of European descents respectively [28, 29]. These findings suggest a racial susceptibility to NAFLD and may have implications for African and other populations and require further research.

### Risk factors for NAFLD

The association of obesity, concurrent hypertension and dyslipidaemia is not surprising and has been well characterized in diabetic populations worldwide. The key pathogenesis of this association is related to insulin resistance and the metabolic syndrome [3, 4]. Our study shows that central obesity and dyslipidaemia although significantly associated with T2DM, are also independent risk factors for NAFLD, more so in men than women. Diabetes and NAFLD have mutual pathogenetic mechanisms, which may interact with metabolic derangements to accelerate NAFLD progression in diabetic patients [10, 11, 25]. The male susceptibility to NAFLD as shown in this study has also been described in Caucasian and Hispanic populations and has been attributed to lifestyle, sex hormones, differing distribution of adiposity, and other behavioral and genetic factors that remain to be fully defined. Although our study identifies important characteristics and associations of NAFLD in this African population, there are a few limitations. The reliance on patient's estimate of their alcohol consumption is subject to recall and may be inaccurate. Additionally, the gold standard for diagnosis of fatty liver is the liver biopsy. This procedure is invasive and was neither feasible nor ethical in this diabetic cohort.

## Conclusion

This study has shown that NAFLD is common in subjects with T2DM and is associated with a 16-fold risk compared to non-diabetic controls. Although obesity, hypertension and dyslipidaemia were also significantly associated with T2DM, our study suggests that central obesity and dyslipidaemiaare also independent risk factors for NAFLD, more so in men than women. Our result supports a role for routine ultrasound scan evaluation in T2DM to enhance clinical decision making and enable early recognition and management of NAFLD to limit its associated adverse hepatic and cardiovascular complications.

### What is known about this topic

- The association of obesity, concurrent hypertension and dyslipidaemia has been well characterized in diabetic populations worldwide;
- In Western countries, patients with diabetes are a high risk group for NAFLD;
- They have a more aggressive clinical course with necro-inflammation and progression to liver disease and enhanced cardiovascular risk.

### What this study adds

- In Nigerians, T2DM is associated with a 16- fold risk of NAFLD compared to non-diabetic controls;
- Nigerian men with T2DM appear more susceptible to NAFLD than women and have a significant association of NAFLD with truncal obesity and dyslipideamia;
- Prevalence of NAFLD in this population is lower than in Caucasians, American blacks, and Hispanics.
